# Targeting activated hepatic stellate cells (aHSCs) for liver fibrosis imaging

**DOI:** 10.1186/s13550-015-0151-x

**Published:** 2015-12-09

**Authors:** Dan Li, Li He, Huizhuang Guo, Hanwei Chen, Hong Shan

**Affiliations:** Department of Radiology, The Third Affiliated Hospital of Sun Yat-sen University, Guangzhou, 510630 China; Guangdong Provincial Engineering Research Center of Molecular Imaging, Guangzhou, 510630 China; Department of Radiology, Guangzhou Panyu Central Hospital, Guangzhou, 511400 China; Interventional Radiology Institute of Sun Yat-sen University, Guangzhou, 510630 China

**Keywords:** Molecular imaging, Activated hepatic stellate cells (aHSCs), Liver fibrosis, Biomarkers, Ligands

## Abstract

Following injurious stimuli, quiescent hepatic stellate cells (qHSCs) transdifferentiate into activated HSCs (aHSCs). aHSCs play pivotal roles in the onset and progression of liver fibrosis. Therefore, molecular imaging of aHSCs in liver fibrosis will facilitate early diagnosis, prognosis prediction, and instruction and evaluation of aHSC-targeted treatment. To date, several receptors, such as integrin αvβ3, mannose 6-phosphate/insulin-like growth factor II receptor (M6P/IGF-IIR), collagen type VI receptor (CVIR), platelet-derived growth factor receptor-β (PDGFR-β), vimentin, and desmin, have been identified as biomarkers of aHSCs. Corresponding ligands to these receptors have also been developed. This review will discuss strategies for developing aHSC-targeted imaging in liver fibrosis.

## Review

### Introduction

Liver fibrosis is a major public health problem and contributes to substantial morbidity and mortality. Iterative injury, abnormal wound healing processes, and redundant extracellular matrix (ECM) accumulation lead to liver fibrosis. Liver fibrosis can be divided into several stages according to the extent of fibrosis. Cirrhosis, an advanced stage of liver fibrosis, can cause many severe complications including portal hypertension, hepatic insufficiency, blood disorders, and hepatocellular carcinoma. Early diagnosis and precise staging of liver fibrosis are very important in managing the disease.

Although liver biopsy is regarded as the gold standard to evaluate liver fibrosis, it has several disadvantages including invasive nature, sampling error, inter/intra-observer variation in the pathological measurement, and the related complications [1, 2]. Multiple serum markers have been employed for liver fibrosis assessment but with limited sensitivity and specificity. Besides conventional imaging techniques, several new imaging techniques, including ultrasound-based transient elastography (TE) [3, 4], magnetic resonance (MR) elastography [5–7], acoustic radiation force impulse (ARFI) ultrasound imaging [8, 9], MR diffusion-weighted imaging (DWI) [10–12], T1*ρ* MR imaging [13–15], and MR perfusion-weighted imaging (PWI) [16, 17], have been applied to detect liver fibrosis. However, these techniques are usually based on morphological alterations of the liver and thus have difficulties to detect liver fibrosis at the early initiation stage or reflect the activity of liver fibrosis accurately. On the contrary, molecular imaging can provide the cellular or molecular information of a diseased liver, which will facilitate early diagnosis and accurate staging of liver fibrosis. In this review, we summarize recent studies on activated hepatic stellate cell (aHSC)-targeted imaging in liver fibrosis.

### Biological and pathological function of hepatic stellate cells

**Fig. 1:**
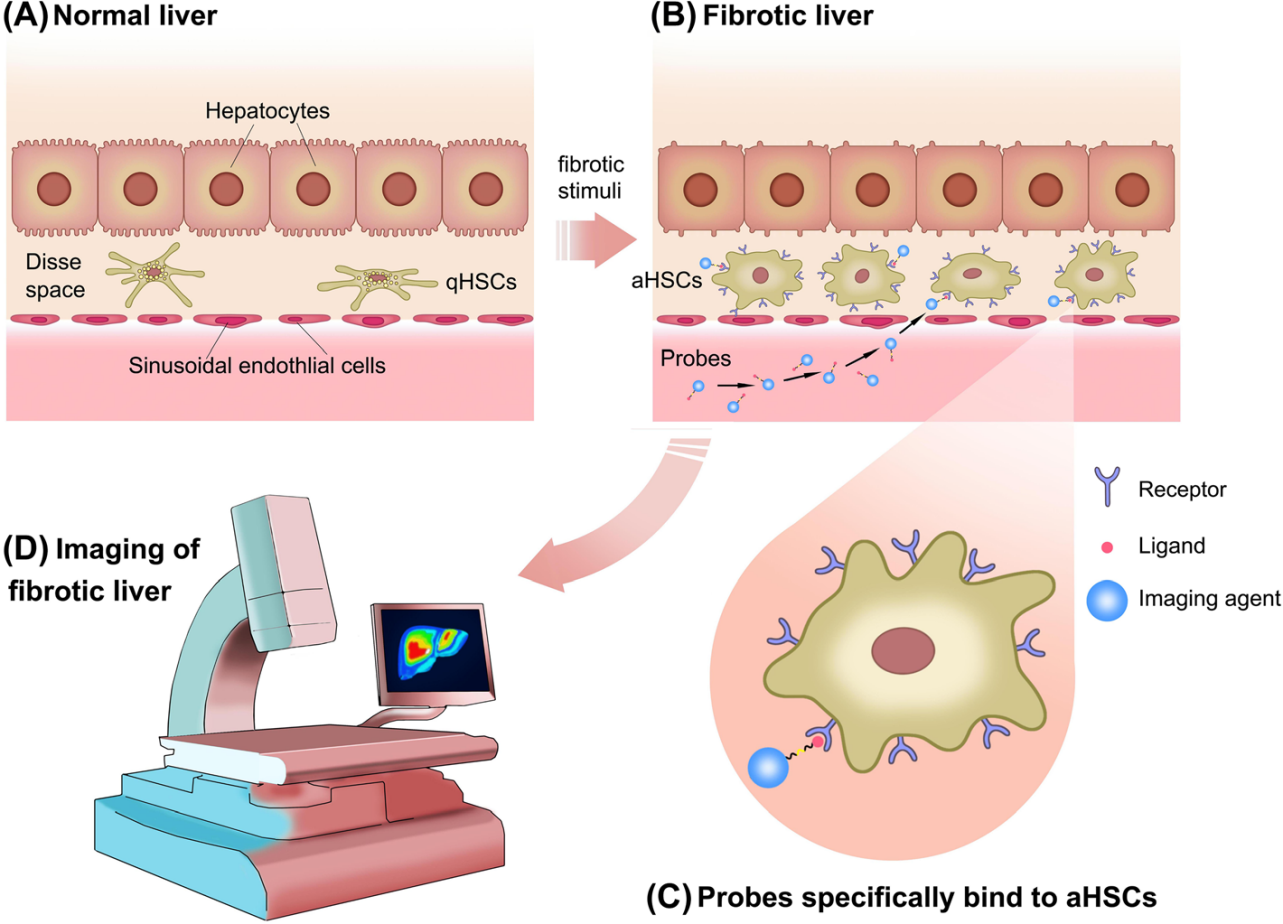
Schematic diagram of activated hepatic stellate cell (aHSCs)-targeted imaging in liver fibrosis. **a** In normal liver, HSCs are in the quiescent state, i.e., quiescent HSCs (qHSCs). **b** Following fibrotic stimuli, qHSCs transdifferentiate into activated HSCs (aHSCs). Receptors that are specifically upregulated on aHSCs are potential targets for molecular imaging of liver fibrosis. **c** Magnified image that demonstrates imaging probes’ specific binding to aHSCs. **d** Imaging of liver fibrosis

Hepatic stellate cells (HSCs) are situated in the space of Disse, between hepatocytes and sinusoidal endothelial cells. They constitute ~15 % of the total liver resident cells [18] and account for ~1.5 % of the total liver volume. In normal liver, HSCs are in the quiescent state and play important roles in supporting liver development and regeneration, vitamin A storage, immunoregulation, liver hemodynamic homeostasis, etc. [19]. Following injurious stimuli, quiescent HSCs (qHSCs) transdifferentiate into aHSCs. HSC activation consists of two main phases: initiation and perpetuation [19, 20]. During the initiation phase, HSCs have gene and phenotype alteration to facilitate cellular response to a range of cytokines. After entering the perpetuation phase, HSCs are characterized by various changes in cell behavior, such as increase in the absolute cell number, ECM production, migration towards chemokines, contraction, loss of retinoid droplets, altered matrix degradation, and inflammatory signaling. aHSC quantity is clearly associated with fibrosis severity [21, 22]. Moreover, resolution of fibrosis is attributed to aHSC apoptosis [23], senescence [24], or their reversion to the quiescent state. Based on their important pathological role, aHSCs are essential targets for the diagnostic imaging of liver fibrosis (Fig. 1). Molecular imaging of aHSCs in liver fibrosis is expected to achieve the following objectives: (1) early diagnosis (aHSC detection before the pathological changes in the liver), (2) prognosis prediction (progression or regression), and (3) instruction and evaluation of aHSC-targeted treatment.

### Targets with imaging

#### Integrin αvβ3

Integrins are heterodimeric glycoprotein receptors formed by α and β subunits. To date, 18 types of α subunits and 8 types of β subunits have been recognized in mammals [25]. Different assemblies of the α and β subunits result in 24 distinct integrins [26], and each type of integrin has a defined binding specificity and signal transduction pathway. Integrins are the major receptors that mediate cellular adhesion and reaction to the ECM and thus play essential roles in regulating cell migration, growth, division, survival, differentiation, and apoptosis. Dysfunction of integrins is found in various pathological processes. Among the integrin family, integrin αvβ3 has been most thoroughly studied. It is highly expressed in both tumor cells [27] and activated endothelial cells [28–30] and regulates tumor progression, metastasis, and angiogenesis. Various ECM proteins like vitronectin, fibrinogen, and fibronectin interact with the integrin αvβ3 via the arginine-glycine-aspartate (RGD) motif [31]. Based on this discovery, diverse RGD derivatives have been developed using many synthetic strategies including RGD-flanking amino acid residues (RGD4C, RGD10) [32, 33], cyclization (cRGDyK, cRGDfK) [34, 35], and N-methylation (cRGDf-N(Me)V) [36]. Several nucleic acid aptamers were also reported to specifically recognize integrin αvβ3 [37–39]. Integrin αvβ3-targeted imaging [40, 41] and therapy [42, 43] in tumor have been extensively studied using these RGD ligands.

**Fig. 2:**
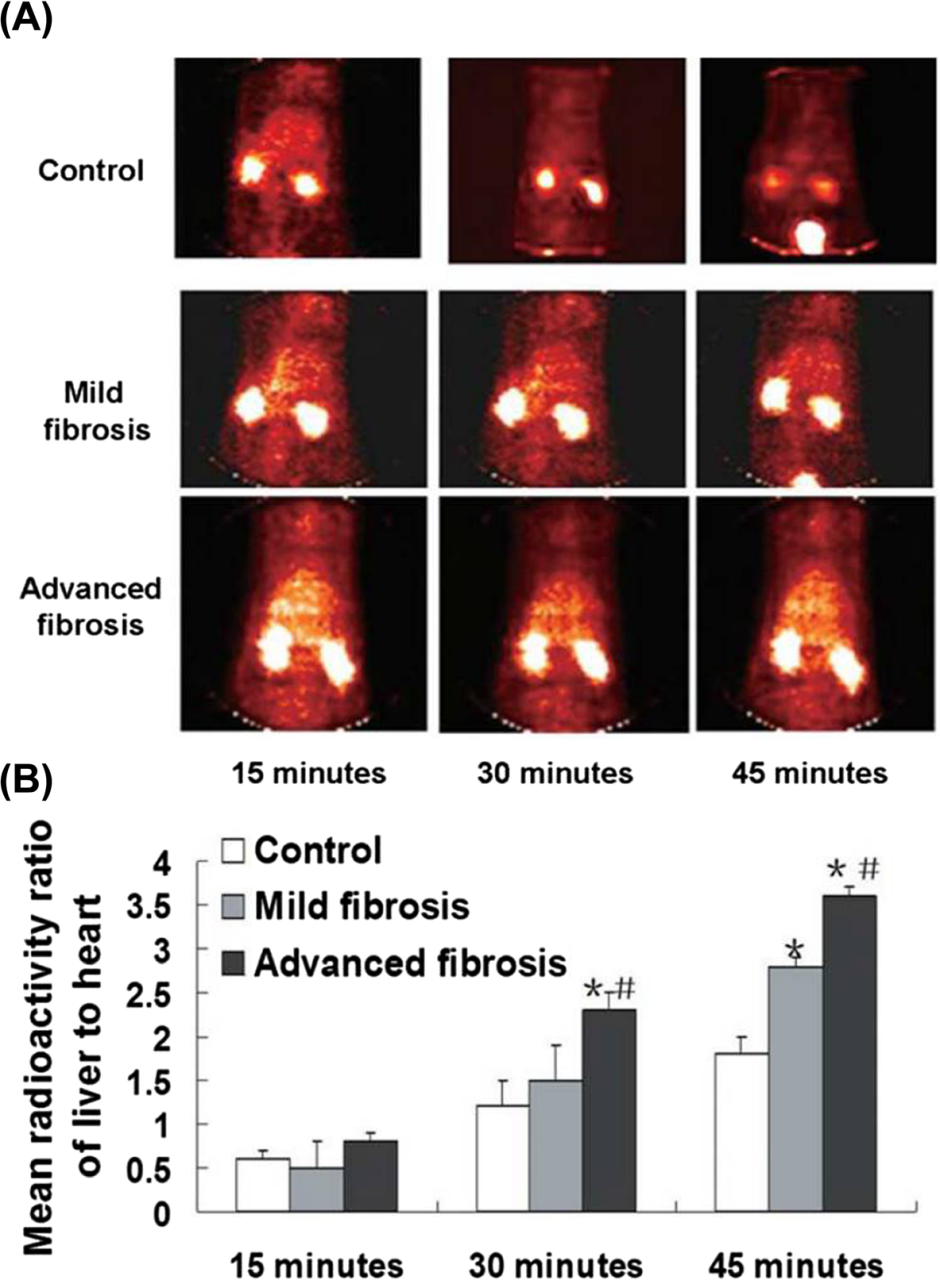
Radionuclide images of the integrin αvβ3 expression in the livers of the normal control and liver fibrosis rats. Mild and advanced fibroses were respectively induced in rats by thioacetamide (TAA) treatment for 3 and 9 weeks. Each animal was administered 6 μCi of ^99m^Tc-cRGDfK by way of the penile vein. **a** The representative radionuclide images were obtained at 15, 30, and 45 min after administration. **b** The region of interest (ROI) in the liver and heart was discriminated, and the radioactivity (counts/pixel) ratio of the liver to heart was calculated and compared. Data represent means ± SD (*n* = 3 per group). *P < 0.05 versus the control group, #*P* < 0.05 versus mild fibrosis. Reproduced with permission from ref. [47]

Studies in liver fibrosis show that integrin αvβ3 is upregulated on aHSCs [44–46] and promotes HSCs survival and proliferation [44]. In contrast, the expression level of integrin αvβ3 is low in qHSCs, hepatocytes, and other nonparenchymal cells [47]. Therefore, integrin αvβ3 can serve as a novel target for molecular imaging of HSCs. Cyclic pentapeptides cRGDyK [34] and cRGDfK [35] are the most exploited for integrin αvβ3 targeting. Cellular experiments demonstrated that cRGDfK was uptaken by aHSCs instead of qHSCs or hepatocytes [45]. ^125^I-cRGDfK-based historadioautography assay of rat hepatic sections showed that the hepatic relative densitometry was positively correlated with the severity of liver fibrosis [47]. Nuclear imaging, a highly sensitive technology, is widely used in both pre-clinical and clinical studies. ^99m^Tc is one of the most popular radionuclides because of its desirable nuclear properties (*t*_1/2_ = 6.02 h, *E*γ = 140.51 keV, *I*γ = 89.06 %), facile availability, and low cost. Li et al. [47] systemically investigated the potential of ^99m^Tc-labeled cRGDfK for single-photon emission computed tomography (SPECT) imaging of HSC activity in fibrotic livers. ^99m^Tc-cRGDfK was administrated through intravenous (i.v.) injection to assess the hepatic expression of integrin αvβ3 in fibrotic (thioacetamide, TAA treatment) and control rats. At 45 min post injection (p.i.), the mean radioactivity ratio of the liver to heart (MRAR) could distinguish among rats with normal, mild fibrotic (TAA treatment for 3 weeks), or advanced fibrotic (TAA treatment for 9 weeks) liver (Fig. 2). ^99m^Tc-cRGDfK uptake in fibrotic liver was blocked successfully through co-administration of cold cRGDfK, which confirmed the specificity of liver uptake. Small peptides are predominantly cleared via the kidney. Besides, integrin αvβ3 is expressed on renal glomerular endothelial cells and, to a lesser extent, on tubular endothelial cells [48, 49]. Therefore, kidney uptake of ^99m^Tc-cRGDfK was high. In this condition, radiotoxicity to the kidneys needs to be considered.

To improve integrin αvβ3-targeted imaging, the binding avidity for integrin αvβ3 has been maximized through the use of dimeric or multimeric cyclic RGD peptides [48, 50–52]. ^99m^Tc-3PRGD2 (3PRGD2 = PEG4-E[PEG4-c(RGDfK)]2; PEG4 = 15-amino-4,7,10,13-tetraoxapentadecanoic acid) is one promising radiotracer [53–55]. The addition of PEG4 linkers increases the distance between the two RGD motifs and thus facilitates simultaneous binding to the neighboring integrin αvβ3 [53]. ^99m^Tc-3PRGD_2_ shows fast excretion kinetics from the liver and kidneys [53, 54], which will lead to better lesion-to-background contrast. Moreover, ^99m^Tc-3PRGD_2_ can be readily produced in high yield and purity from a kit formulation [54, 55]. In clinical trials, ^99m^Tc-3PRGD2 imaging is sensitive for cancer detection [55, 56]. Zhang et al. further used ^99m^Tc-3PRGD2 for a liver fibrosis study [57]. At 30 min p.i., the MRAR in rats with advanced liver fibrosis (1.98 ± 0.08) was significantly higher than that in control rats (1.50 ± 0.12). Also, the liver *t*_1/2_ in the fibrosis group (27.07 ± 10.69 min) was significantly longer than that in the control group (12.67 ± 4.10 min). However, the researchers did not study whether ^99m^Tc-3PRGD2 could be used for fibrosis staging. In both of the above two studies [47, 57], clinical SPECT machines were used for imaging; thus, the MRAR was relatively low and should be improved to attain precise diagnosis. Since ^99m^Tc-3PRGD2 has the potential for clinical translation, clinical trials in patients with liver fibrosis are also expected.

**Fig. 3:**
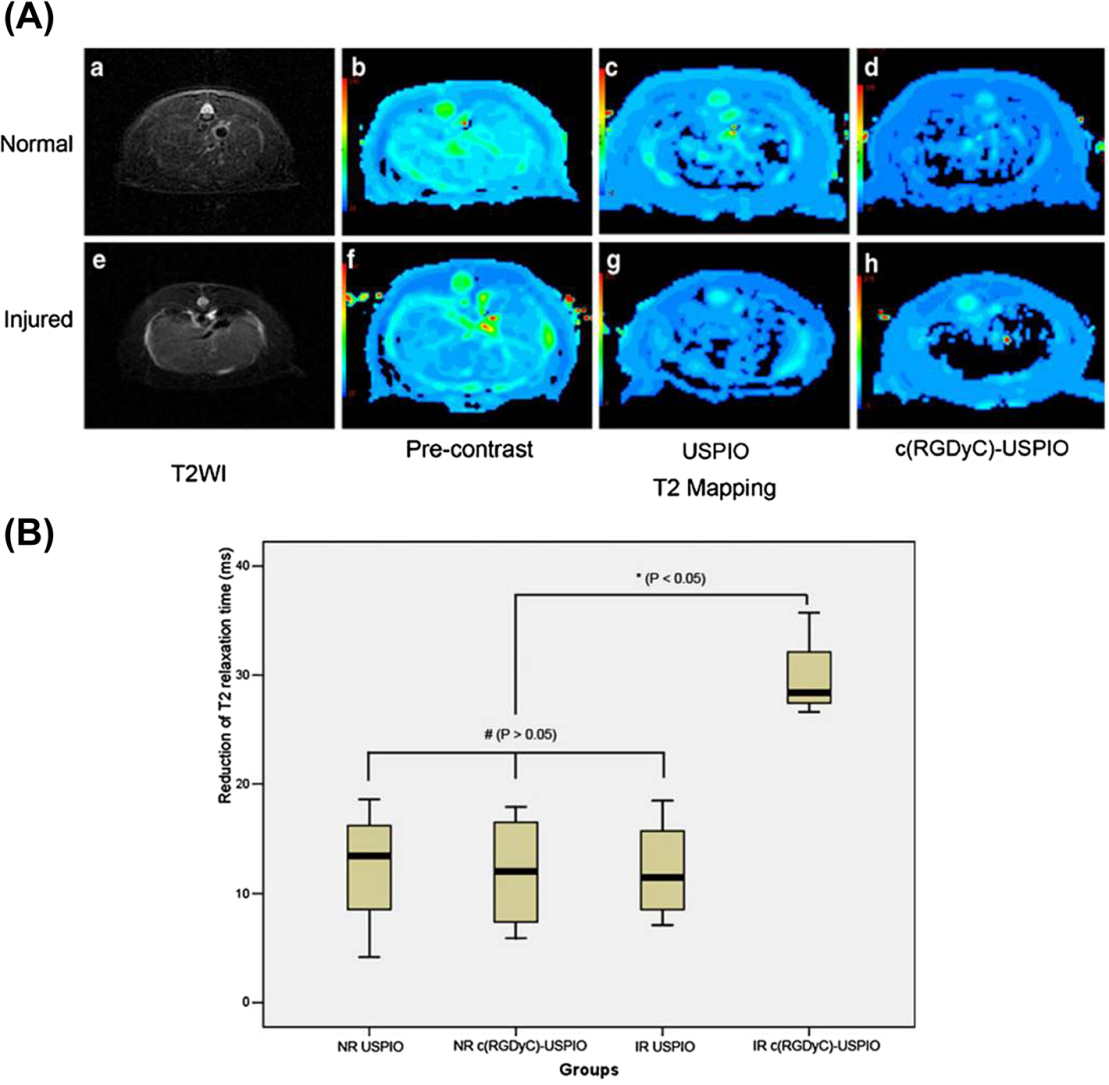
MR images of the αvβ3 integrin expression in the livers of the normal control and liver fibrosis rats. **a** MR imaging studies in normal rats (*NR*) and injured rat (*IR*, with early-staged liver fibrosis, CCl4 treatment for 3 weeks) after administration of USPIO or cRGDyC-USPIO. **b** The reduction of T2 relaxation times after the administration of USPIO or cRGDyC-USPIO in the normal and injured rat groups. Reproduced with permission from ref. [46]

Magnetic resonance (MR) imaging produces images using magnetic fields and radio waves. It is absent of radiation and excellent at providing both anatomic and functional information. Both T1-positive (e.g., gadolinium chelates) and T2-negative (e.g., superparamagnetic iron oxide nanoparticles) contrast agents are used for MR imaging to boost up imaging sensitivity. Wang et al. conjugated cRGDyC with ultrasmall superparamagnetic iron oxide (USPIO) for aHSC-targeted MR imaging [46]. The preparation of the cRGDyC-USPIO probe includes three steps: synthesis of USPIO coated with oleic acid; surface coating with 1,2-distearoyl-sn-glycero-3-phosphoethanolamine-*N*-[carboxy(polyethylene glycol)-2000 (DSPE-PEG)] and 1,2-distearoyl-sn-glycero-3-phosphoethanolamine-*N*-[maleimide(polyethylene glycol)-2000 (DSPE-PEG-Mal)]; and cRGDyC conjugation to the nanoparticles. cRGDyC-USPIO was 13 ± 3 nm in diameter. After administration of cRGDyC-USPIO or USPIO, MR imaging was performed in control rats and rats with early-staged liver fibrosis (CCl4 treatment for 3 weeks) using a clinical 1.5 Tesla (T) scanner. At 4 h p.i., liver T2 relaxation times of fibrosis rats treated with cRGDyC-USPIO decreased significantly compared to those of normal rats with cRGDyC-USPIO, normal with USPIO, and fibrosis with USPIO (Fig. 3). Tissue assay confirmed that cRGDyC-USPIO could specifically target aHSCs. Iron oxide-based T2 imaging not only has the advantage of high sensitivity but also has two major disadvantages: negative contrast effects and artifacts caused by magnetic susceptibility [58]. On the contrary, paramagnetic material-based T1 imaging exerts a bright signal enhancement and has superior spatial resolution [59]. T1-T2 dual-modal MR imaging can combine the strength of each modality and thus offer more accurate information [60]. aHSC-targeted T1-T2 dual-modal MR imaging studies are expected in the future.

#### Vimentin and desmin

**Fig. 4:**
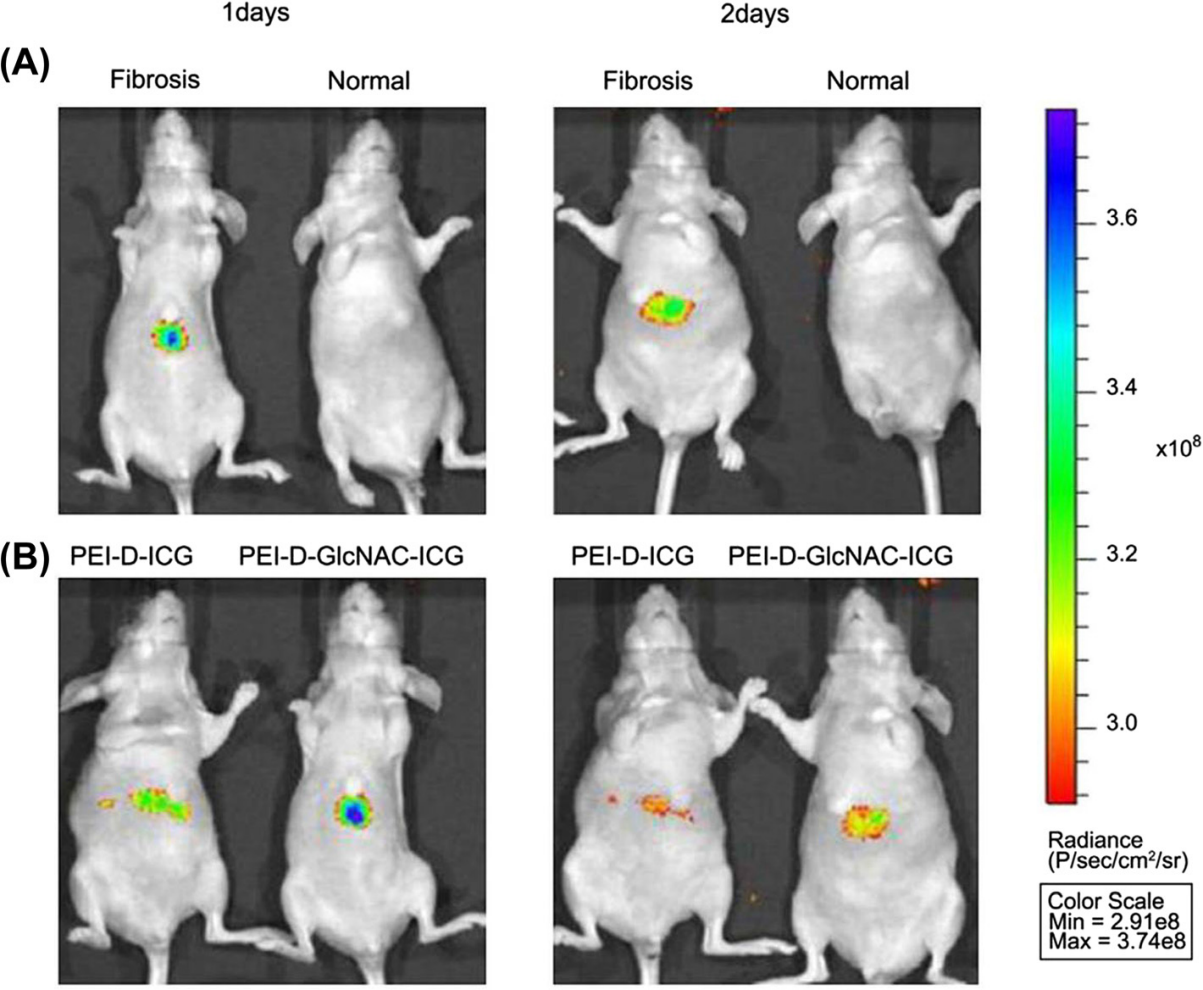
Optical images of the vimentin and desmin expression in the livers of the normal control and liver fibrosis rats. Optical image of fibrotic and normal mice after administering PEI-D-GlcNAc-ICG (**a**) and image of fibrotic mice after injection of PEI-D-ICG and PEI-D-GlcNAc-ICG (**b**). Reproduced with permission from ref. [67]

Both vimentin and desmin belong to the type III intermediate filament protein family and play important roles in maintaining the stability of cellular structure. Besides being distributed in the cytoplasm, these proteins are also recruited to the cell surface in pathological conditions [61–64]. During HSC activation, the expression of both vimentin and desmin is strongly upregulated [65]. *N*-acetylglucosamine (GlcNAc) was identified as a specific glycoside ligand to vimentin and desmin and bound to the rod II domain of these proteins on plasma membrane surfaces [64]. Further study showed that GlcNAc-bearing polymers could bind to freshly isolated HSCs and suppressed cellular activation during in vitro culture [66]. In another study, GlcNAc was conjugated to indocyanine green (ICG) and polyethyleneimine (PEI)/TGFβ1 siRNA (PEI-D-GlcNAc-ICG/siRNA) for liver fibrosis imaging and therapy [67]. Optical imaging was carried out to monitor the distribution of the complexes (Fig. 4). At 1 day p.i., the complexes were retained in fibrotic livers, whereas they had been cleared out in normal livers. Moreover, more PEI-D-GlcNAc-ICG/siRNA was distributed in fibrotic livers compared to the control complex that was absent of GlcNAc ligand. Tissue analysis showed that 79 % of the PEI-D-GlcNAc-ICG/siRNA complex targeted to HSCs. In comparison, only 32 % of the control complex targeted to HSCs. These results imply that GlcNAc could be a valid ligand for aHSC targeting. However, in the above study, imaging was performed at late time points (1 day). To facilitate clinical application, GlcNAc-based imaging is expected to be optimized for liver fibrosis detection at early time points after probe injection. In addition, the linear heptapeptide VNTANST was identified as a specific ligand that recognized vimentin on the cell surface [68].

### Targets without imaging (future work)

#### Mannose 6-phosphate/insulin-like growth factor II receptor (M6P/IGF-IIR)

Mannose 6-phosphate/insulin-like growth factor II receptor (M6P/IGF-IIR) is a 300-kDa single-chain transmembrane glycoprotein. Fifteen repeating domains constitute its large extracytoplasmic region. M6P/IGF-IIR binds to three types of ligands: IGF-II, the M6P-bearing proteins, and retinoic acid. One molecule of M6P/IGF-IIR binds one molecule of IGF-II and two molecules of M6P [69, 70]. IGF-II and M6P have their respective binding sites, but there is a mutual inhibition between these two ligands [71]. M6P/IGF-IIR carries out various functions, including lysosomal protein sorting and growth regulation. In normal liver, qHSCs express few M6P/IGF-IIR. But the receptor is upregulated on the plasma membrane of aHSCs during liver fibrosis [72, 73]. At the cell membrane, M6P/IGF-IIR can bind to transforming growth factor-β (TGF-β) complex via M6P, convert latent TGF-β into active TGF-β [72, 74], and thus promote fibrogenesis.

In 1999, Beljaars et al. took the lead to demonstrate that human serum albumin (HSA) modified with M6P could be taken up by aHSCs in fibrotic livers [75]. When 28 molecules of M6P were coupled to 1 molecule of HSA (M6P_28_-HSA), the hepatic accumulation increased to 59.2 ± 9.2 % in fibrotic rats and M6P_28_-HSA was preferentially uptaken by aHSC. This drug carrier (M6P-HSA) has been used to cargo therapeutic compounds to aHSCs in liver fibrosis [76–79], leading to enhanced drug efficacy and minimized drug toxicity. To date, M6P/IGF-IIR-targeted aHSC imaging has not been reported and thus is expected in the future. Besides, alternation of phosphate group in M6P with phosphonate, carboxylate, or malonate groups leads to improved binding affinity and stability [80–83]. These analogs could be used to facilitate aHSC targeting.

#### Collagen type VI receptor (CVIR)

Collagen type VI (CVI) is a heterotrimeric glycoprotein composed of three different α chains, α1(VI), α2(VI), and α3(VI) [75]. α3(VI) chains can be substituted by α4(VI), α5(VI), and α6(VI) chains [84]. In cytoplasm, CVI monomers are assembled into dimmers and subsequently into tetramers. End-to-end alignment of secreted tetramers forms microfibrils in ECM [85]. CVI stimulates cell growth, promotes cell survival, and modulates matrix homeostasis through interaction with cells and other matrix molecules [86]. HSCs are the major cells that produce CVI in the liver [87]. CVI is mainly distributed in the portal areas of normal livers. When liver fibrosis occurs, the accumulation of this type of collagen is enhanced, particularly in the fibrous septa [88, 89]. CVI can bind to many types of receptors including integrins α1β1, α2β1, and α1β1 [90–92] and neuron/glia-type 2 (NG2) [93–95]. There are several RGD sequences in CVI, but the cyclic octapeptide C*GRGDSPC* selectively antagonizes the binding of CVI to cells [96]. The specific type of CVI receptor (CVIR) that mediates the attachment of this peptide to cells has not been defined.

HSA modified with 10 C*GRGDSPC* moieties (pCVI-HSA) was demonstrated as a carrier specifically targeting aHSCs [97]. Cellular experiments showed that aHSCs uptook much more pCVI-HSA compared to qHSCs. This implies that CVIR is upregulated on aHSCs. In fibrotic livers, aHSCs were the principal cells that bound the carrier. The cyclization of C*GRGDSPC* is accomplished via disulfide bond generation between two adjacent cysteine residues. A further modification was made to the peptide by substituting lysine for cysteine which resulted in C*GRGDSPK* [98, 99]. The modified peptide is cyclized through an amide linkage between the cysteine and lysine residues and thus is more stable. This peptide was conjugated to liposomes for aHSC-targeted drug delivery in liver fibrosis [98, 99]. aHSC-targeted imaging based on this kind of peptide is anticipated in the future studies.

#### Platelet-derived growth factor receptor-β (PDGFR-β)

The platelet-derived growth factor (PDGF) is one of the most extensively investigated growth factors. In liver fibrosis, PDGF contributes to several behavior changes of HSCs in the process of activation, including proliferation, migration towards chemokines, and loss of retinoid droplets [100]. The PDGF family contains five dimeric members (PDGF-AA, PDGF-AB, PDGF-BB, PDGF-CC, and PDGF-DD) derived from four distinct polypeptide chains (PDGF-A, PDGF-B, PDGF-C, PDGF-D) [100, 101]. PDGF-A and PDGF-B are secreted in an active form, whereas PDGF-C and PDGF-D demand extracellular proteolytic activation after being secreted. PDGF members exert their actions through binding to two different receptors, PDGF receptor PDGFR-α and PDGFR-β. PDGFR-α binds to PDGF-AA, PDGF-AB, PDGF-BB, and PDGF-CC, while PDGFR-β binds to PDGF-BB and PDGF-DD [101]. In qHSCs, there is a constitutive expression of PDGFR-α, whereas PDGFR-β expression is not detected [102]. The expression level of PDGFR-β is significantly increased on aHSC [102, 103].

Arginine-27 and isoleucine-30 in the PDGF-B chain are crucial for receptor binding [104]. Based on this work, Beljaars et al. designed a cyclic peptide (C*SRNLIDC*) that recognized PDGF receptors [105]. A targeted drug carrier was further produced through covalently linking 15 C*SRNLIDC* moieties to 1 HSA moiety (pPB-HSA) [105]. In vitro studies demonstrated that the cellular uptake of pPB-HSA in aHSCs was significantly higher than that in qHSCs. After i.v. injection, the majority of pPB-HSA was localized in aHSCs of fibrotic livers. C*SRNLIDC* has been applied to aHSC-targeted liver fibrosis therapy using HSA or liposomes as drug delivery vehicles [106–108]. The linear tridecapeptide ANFLVWEIVRKKP [109] and cyclic PDGF-BB^73–81^ (R*KIEIVRKKC*) [110, 111] have also been identified as a PDGF-BB analog that recognized PDGF receptors. Although the PDGF-B chain is a ligand to both types of PDGFR, its asparagine-117 and leucine-119 are principally critical for PDGFR-β binding [112]. Therefore, it is possible to design PDGF-BB analogs which exclusively bind to PDGFR-β. Besides, a PDGFR-β-specific RNA aptamer was reported recently [113]. Application of the above ligands to aHSC-targeted imaging remains to be investigated.

### Future prospects

Several factors should be considered when designing imaging probes for aHSCs. First, the liver is regarded as the second most complex organ. Other cell types of the liver, such as Kupffer cells, sinusoidal endothelial cells, and hepatocytes, may nonspecifically uptake the probes. High molecular weight proteins, like serum albumin, are mainly metabolized by the liver. Although serum albumin-based carriers (M6P_21_-BSA, pCVI-HSA, pPB-HSA) preferentially targeted HSCs in fibrotic livers, they were uptaken by endothelial cells [75, 97] or hepatocytes [105] in normal livers. Therefore, the accumulation level of these carriers in both fibrotic and normal livers was similar [75, 97, 105]. This characteristic makes these carriers suitable for HSC-targeted drug delivery rather than imaging. Low molecular weight ligands (such as peptides, aptamers), which are cleared mainly through the kidneys, will be more appropriate for this kind of imaging. Besides, the addition of PEG to probes could decrease the nonspecific uptake by Kupffer cells [46]. Second, novel MR techniques including MR elastography [5–7], MR DWI [10–12], T1*ρ* MR imaging [13–15], and MR PWI [16, 17] have emerged for detecting liver fibrosis. A combination of these techniques with aHSC-targeted MR imaging could provide abundant disease information on anatomical, functional, and molecular levels. Nuclear imaging techniques, including SPECT and positron emission tomography (PET), are often used for tumor imaging [114]. They not only have high sensitivity but also cause radio damage. Therefore, the pros and cons should be weighed before applying these techniques to liver fibrosis diagnosis. Third, ultrasound imaging has the advantages of high soft tissue contrast, low cost, and no radiation. Various kinds of bubbles have been developed as ultrasound contrast agents [115]. Among them, nano-sized bubbles, which can extravasate from blood vessels, are more suitable for imaging of extravascular cells. Thus, aHSC-targeted ultrasound imaging could potentially be accomplished through conjugating specific ligands to nanobubbles. Four, recent studies imply the bidirectional crosstalk between aHSCs and tumor cells [116, 117]. Tumor-derived factors activate HSCs, and in turn, aHSCs promote phenotypic changes, proliferation, and invasion of tumor cells. Therefore, aHSC-targeted imaging in liver cancers could help better understand the pathophysiology of the tumor microenvironment and further instruct therapy.

## Conclusions

HSC activation plays pivotal roles in the onset and progression of liver fibrosis. Receptors, such as integrin αvβ3, M6P/IGF-IIR, CVIR, PDGFR-β, vimentin, and desmin, have been identified as biomarkers of aHSCs. Corresponding ligands to these receptors have also been developed (summarized in Table 1). Many studies focused on aHSC-targeted drug delivery for the treatment of liver fibrosis through taking advantage of these ligands. However, to our knowledge, only a few studies targeted aHSCs for in vivo imaging. To facilitate clinical translation, further studies are expected to optimize imaging probes for aHSCs.

**Table 1:** aHSCs biomarkers and corresponding ligands (*aHSC-targeted imaging studies)

Biomarker	Ligand	Reference
Integrin αvβ3	cRGDfK	[35, 45, 47*, 57*]
	cRGDyC	[46*]
	RGD4C (ACDC**RGD**CFCG)	[32]
	RGD10 (DGARYC**RGD**CFDG)	[33]
	cRGDf-N(Me)V	[36]
	Apt-αvβ3-1	[37]
	(5'-GGGAGACAAGAAUAAACGCUCAAUUCAACGCUGUGAAGGGCUUAUACGAGCGGAUUACCCUUCGACAGGAGGCUCACAAAAGGC-3')	
	Apt-αvβ3-2	[38]
	(5′-UUCAACGCUGUGAAGGGCUUAUACGAGCGGAUUACCC-3′)	
	Apt-αvβ3-3	[39]
	(5′- AGTTCGZZZZAAGAAAZZAGCACACCGZZGACZZGZZZAGZGGCGGACCA-3′)	
	Z: 5-N-(benzylcarbox-yamide)-2′-deoxyuridine	
Vimentin and desmin	*N*-acetylglucosamine (GlcNAc)	[64, 66, 67*]
	VNTANST	[68]
M6P/IGF-IIR	M6P	[75]
	Phosphonate, carboxylate, or malonate analogs of M6P	[80–83]
CVIR	C*GRGDSPC*	[96, 97]
	C*GRGDSPK*	[98, 99]
PDGFR-β	C*SRNLIDC*	[105]
	ANFLVWEIVRKKP	[109]
	PDGF-BB^73–81^	[110, 111]
	(R*KIEIVRKKC*)	
	Apt-PDGFR-β	[113]
	(5′-UGUCGUGGGGCAUCGAGUAAAUGCAAUUCGACA-3′)	

### Ethical approval

This article does not contain any studies with animals or human participants performed by any of the authors.
